# Development and Characterization of Spray-Dried Curcumin–Lecithin Complexes with Improved Solubility and In Vitro Digestive and Thermal Stability

**DOI:** 10.3390/foods14183157

**Published:** 2025-09-10

**Authors:** Erkan Mankan, Osman Sagdic, Ayse Karadag

**Affiliations:** 1 Department of Food Engineering, Faculty of Chemical and Metallurgical Engineering, Yildiz Technical University, Davutpasa, Istanbul 34210, Turkey; erkan.mankan@trustlifeventures.com (E.M.); osagdic@yildiz.edu.tr (O.S.); 2 Trustlife Labs Drug Research & Development Center, Istanbul 34774, Turkey

**Keywords:** phytosome, maltodextrin, TEM, SEM, simulated digestion

## Abstract

Curcumin, a bioactive polyphenol from turmeric, faces significant challenges in food and pharmaceutical applications due to its poor water solubility, low stability, and limited bioavailability. In this study, curcumin–lecithin complexes (phytosomes) were spray-dried using maltodextrin as the carrier polymer to produce free-flowing powders with improved physicochemical properties. The powders were characterized based on moisture content, particle size, morphology, curcumin loading, thermal behavior, and stability under simulated gastrointestinal and thermal conditions. The lecithin–curcumin complexes exhibited high entrapment efficiency (up to 94%), a predominantly amorphous structure, and improved thermal and digestive stability compared to free curcumin. Particle size and wettability were influenced by carrier and curcumin ratios, with maltodextrin enhancing powder flowability and apparent solubility. Morphological analyses revealed spherical particles with core–shell structures, confirming successful complexation. The complexes protected curcumin from degradation at intestinal pH and elevated temperatures, highlighting their potential for enhanced bioavailability. These findings demonstrate that spray-dried curcumin–lecithin complexes with maltodextrin carriers offer a promising strategy to overcome curcumin’s solubility and stability limitations, supporting their application in functional foods and pharmaceuticals.

## 1. Introduction

Curcumin, a yellow polyphenol compound extracted from the rhizome of turmeric (*Curcuma longa*), has been the subject of extensive research over the past three decades due to its remarkable health benefits in preventing and treating various pro-inflammatory chronic diseases. Structurally, curcumin is a symmetrical diferuloylmethane molecule characterized by an ordered crystalline arrangement, a chemical formula of C_21_H_20_O_6_, and a molecular weight of 368.385 g/mol. The melting point (T_m_) and thermal degradation temperatures of curcumin depend on measurement conditions and data processing. The melting point of curcumin typically ranges from 172 to 187 °C [1,2,3]. Thermal degradation temperatures, measured using thermogravimetric analysis, range from 224.11 to 382.66 °C at a heating rate of 10 °C/min and from 205 to 441 °C at a heating rate of 20 °C/min [4,5,6,7]. Three distinct crystalline forms of curcumin have been identified: monoclinic form I and orthorhombic forms II and III. Among these, monoclinic form I is the most thermodynamically stable, attributed to strong intermolecular O–H···O bonding. Orthorhombic forms, with weaker hydrogen bonding networks, present higher water solubility and faster dissolution rates in aqueous media but require labor-intensive and energy-consuming processes for production, limiting their commercial scalability [8]. Forms II and III of curcumin exhibit higher crystal densities compared to form I; however, their melting endotherms occur at lower temperature in differential scanning calorimetry analysis [9]. The melting points of curcumin polymorphs obtained through crystallization were reported as follows: T_m_ (peak) of form I was 181.42 °C, while forms II and III showed melting points of 175.12 and 172.85 °C, respectively [10]. Additionally, melting points of 179, 176, and 173 °C for forms I, II, and III, respectively, were reported for polymorphs obtained using solvent evaporation under different reduced pressures and with liquid antisolvent precipitation methods [8]. Commercial curcumin predominantly exists as form I. Additionally, curcumin is classified as a good glass former (Class III), as evidenced by the absence of detectable crystallization during cooling and subsequent reheating cycles [11].

Curcumin demonstrates moderate lipophilicity, as indicated by a log *p* value of 3.2–4.1, and exhibits very poor aqueous solubility (approximately 11 ng/mL at 25 °C), though it becomes more soluble under alkaline conditions. It possesses three dissociable acidic protons, with corresponding pKa values of 7.8, 8.5, and 9.0. Above neutral pH, curcumin rapidly undergoes hydrolytic degradation, with a half-life of less than 30 min, yielding various breakdown products such as trans-6-(4′-hydroxy-3′-methoxyphenyl)-2,4-dioxo-5-hexanal, ferulic acid, feruloylmethane, and vanillin [12].

The poor water solubility of hydrophobic bioactives such as curcumin poses a substantial challenge for oral bioavailability, particularly in the aqueous environment of the gastrointestinal (GI) tract. Absorption of curcumin necessitates its prior solubilization into mixed micelles during digestion, followed by transport across the epithelial cells lining the GI tract. Only the fraction of curcumin that becomes bioaccessible—capable of moving through GI fluids, penetrating the mucus layer, and being absorbed by the enterocytes—can ultimately reach systemic circulation [13].

Numerous studies have reported very low or even undetectable plasma concentrations of curcumin following oral administration, highlighting its limited bioavailability. This is primarily attributed to a combination of factors, low aqueous solubility, chemical instability (such as rapid hydrolytic degradation), rapid metabolic transformation, and low permeability across the intestinal cell membrane, as demonstrated by an apparent permeability coefficient (*P*_app_, apical to basolateral) value of 2.93 ± 0.94 × 10^−6^ cm/s in the Caco-2 cell model, with no evidence of active efflux during transport [14,15]. Moreover, curcumin is highly susceptible to degradation by environmental factors such as heat and light, further restricting its applications within food and pharmaceutical industries [16].

The overall bioavailability of bioactive compounds like curcumin depends on their transport pathway to systemic circulation—via either the portal vein or the lymphatic system. Compounds absorbed through the portal vein undergo extensive enzymatic metabolism in the liver (first-pass metabolism), thereby substantially reducing their systemic availability. Conversely, lymphatic transport bypasses the liver, preserving greater portion of the bioactive compound. Moderately lipophilic compounds such as curcumin (Log D_7_._4_ < 5) typically follow the portal vein route, resulting in significant losses from first-pass metabolism. Clinical studies have demonstrated that the absorption and bioavailability of curcuminoids are increased when administered in food matrices, particularly those containing suspended lipids or polar lipid components [17]. During intestinal lipid digestion, free fatty acids and monoacylglycerols released from digestible lipids increase the solubilization capacity of mixed micelles in the small intestine, thereby facilitating curcumin’s transport via the lymphatic system rather than the portal vein [13,18]. Additionally, the concurrent use of adjuvants that inhibit detoxifying enzymes involved in curcumin metabolism (such as UDP-glucuronosyltransferase and hepatic aryl hydrocarbon hydroxylase) can markedly improve oral bioavailability. For example, co-administration of curcumin with black pepper piperine, quercetin, or silibinin has been shown to significantly increase therapeutic serum concentrations [19].

Beyond the strategy of co-digesting curcumin/turmeric powder with properly formulated foods specifically, encapsulating within suitable delivery systems has also proven highly effective in improving bioavailability. A variety of delivery systems including micelles, emulsions, solid lipid nanoparticles, oil bodies, hydrogels, liposomes, and nanoparticles have been extensively evaluated [13,20,21,22,23,24]. Among these, phospholipid complexes (phytosomes) have emerged as particularly promising, significantly enhancing solubility, membrane permeability, chemical stability, and biological efficacy of polyphenols, as reviewed in previous studies [25,26,27,28,29].

Various research findings suggest that phospholipid–curcumin complexes represent one of the most promising approaches to enhance curcumin stability and bioavailability [30,31]. The formation of these complexes could improve curcumin’s pharmacokinetic profile by protecting it from retro-Claisen fragmentation, the primary degradation pathway in aqueous systems [32]. Multiple studies, including randomized, double-blind, crossover human trials, have demonstrated a significant increase in bioavailability following oral administration of curcumin–phospholipid complexes [19,33,34].

Phospholipids like lecithin are amphiphilic molecules composed of a hydrophilic head (e.g., choline phosphates) and hydrophobic tails (long hydrocarbon chains). They are widely employed as emulsifiers in the food industry without restrictions on their maximum allowable levels. In addition to their functional role, phospholipids offer numerous health benefits such as hepatoprotection, cholesterol clearance, vascular disease prevention, and cognitive improvements. As principal components of cell membranes, phospholipids can penetrate mammalian cell membranes and enter the cytoplasm without disturbing the lipid bilayer, enabling them to safely and effectively enhance the bioavailability of active compounds [35,36]. The interaction between polyphenols and phospholipids is primarily driven by hydrogen bonding between the hydroxyl groups of polyphenols and the phosphate and choline groups of phospholipids, along with hydrophobic interactions. In such complexes, the hydrophobic tails of phospholipids aggregate to form a hydrophobic cavity where hydrophobic polyphenol binds through hydrophobic interactions, while the hydrophilic portions face outward [37,38]. Upon entering the intestinal tract, the amphipathic nature of these complexes facilitates their transition from the aqueous environment of the intestinal lumen to the lipid-soluble environment of the enterocyte cell membranes [30].

Several methods have been developed for phytosome preparation, including solvent evaporation, anti-solvent precipitation, and more recently supercritical fluid methods [38,39,40]. Typically, phospholipid and polyphenol are mixed in stoichiometric ratios ranging from 1:1 to 10:1 and dissolved in either aprotic (e.g., ethyl acetate, methylene chloride, aromatic hydrocarbons) or protonic solvents (e.g., ethanol, methanol). After precipitation and solvent removal under reduced pressure, the residue is dried by freeze-drying or vacuum-drying [41,42,43]. Nevertheless, the sticky nature of phospholipid complexes poses challenges for industrial processing, storage, and dissolution. To address this, carrier substances such as fumed silica and hydroxyapatite have been incorporated into the polyphenol–phospholipid mixture before solvent evaporation, resulting in free-flowing powders with well-dispersed structures [39,44,45]. Additionally hydrophilic polymers like sodium alginate, carboxymethyl cellulose, and PVP (polyvinylpyrrolidone) have been used as stabilizers for curcumin–lecithin complexes, where polymers and lecithin are dissolved in water and combined with curcumin dissolved in acetone, followed by ultrasonication, solvent evaporation, and freeze-drying [46].

Spray-drying has proven effective for converting aqueous liposomes [47,48], and oil-in-water emulsions into dried powders using carriers such as maltodextrin, gum Arabic, Konjac glucomannan, whey protein isolate, and mannitol [21,49]. In a study by Nguyen et al. [50], lecithin–curcumin particles were produced via spray-drying using gum Arabic, an expensive surface-active polysaccharide, at relatively low lecithin-to-curcumin mass ratio (20:1), which yielded 0.8–2.4% curcumin in the final powder due to the mass of the carrier.

In this study, spray-drying was employed to both evaporate solvent (ethanol) from lecithin–curcumin complexes and generate free-flowing phytosomal powders using maltodextrin (MD), a cost-effective, non-surface-active, and widely used carrier. Lecithin-to-curcumin mass ratios of 2.5:1 and 5:1, along with carrier to-lecithin–curcumin complex ratios of 2:1 and 0.7:1, were adopted to achieve a high curcumin loading (8–14%) in the final powders. The resulting phytosomal curcumin powders were systematically characterized in terms of yield and key physicochemical attributes, including moisture content, water activity, color, dissolubility, wettability, density, particle size distribution, curcumin loading (%), morphology (SEM and TEM), Fourier-transform infrared (FT-IR) spectra, dynamic scanning calorimetry (DSC), and X-ray diffraction (XRD), as well as thermal and in vitro digestive stability.

## 2. Materials and Methods

Curcumin (≥95% purity, HPLC) was obtained from JIAHERB, Inc. (Xi’an, China). Maltodextrin (Glucidex^®^ 19) was obtained from Roquette Frères (Lestrem, France). Soy lecithin (Lipoid P75, phospholipids from soybean containing ~70% phosphatidylcholine, non-GMO) was obtained from Lipoid GmbH (Ludwigshafen, Germany). Erythromycin (≥98% purity), chlorambucil (≥98% purity), pepsin from porcine gastric mucosa (600 U/mg, Cat. No. P6887), and trypsin from porcine pancreas (Cat. No. T1426) were obtained from Sigma-Aldrich (St. Louis, MO, USA). Dimethyl sulfoxide (DMSO), acetonitrile (HPLC grade), methanol (HPLC grade), and other chemicals and reagents were of analytical grade and obtained from Sigma-Aldrich (St. Louis, MO, USA), unless otherwise stated. Pure and ultrapure water used in all experiments was prepared using Geno™ II and Alto™ I (Avidity Science, Buckinghamshire, UK).

### 2.1. Spray Drying of Curcumin–Lecithin Complexes

The complex was prepared by mixing lecithin and curcumin at a defined ratio (Table 1) and dissolving this mixture in 50% aqueous in ethanol, followed by magnetic stirring for 15 min. The ethanol-to-water ratio of 50:50 (*v*/*v*) was selected based on its ability to simultaneously dissolve lecithin, curcumin, and maltodextrin (MD). Higher alcohol concentrations prevented MD dissolution, while lower concentrations did not dissolve curcumin. MD was added and dissolved in this mixture using magnetic stirring for an additional 30 min and an Ultra-turrax homogenizer at 10,000 rpm for 10 min.

The lecithin free-feed solutions (MD5 and MD15) had 1% of curcumin (*w*/*v*) and 5 and 15% of MD (*w*/*v*) and were also produced under the same conditions. The feed solutions (F1, F2, F3) had 5% of lecithin (*w*/*v*) and varying concentration of MD (5 and 15%, *w*/*v*) and curcumin (1 and 2%, *w*/*v*) (Table 1). All feed solutions were subjected to a laboratory scale spray dryer (Labultima, LUPTPL 228 ADVANCED 2021, Mumbai, India) equipped with solvent converter set at 1 °C (to condense the evaporating solvent). The spray drying parameters were an aspiration rate of 120 m^3^/h, feed flow rate of 600 mL/h, and an air inlet temperature of 170 °C, yielding an outlet temperature of 45 °C. Samples were stored in the dark in the air-tight containers at 4 °C until analysis.

### 2.2. The Yield, Moisture Content, Water Activity, and Color Analysis

Spray-drying yield was calculated as the mass ratio of spray-dried powder to the total solid in the feed. To determine the moisture content, 1 g of each sample was placed in pre-weighed petri dishes and dried at 105 °C for 6 h until the constant weights were reached. The samples were allowed to cool in a desiccator to the room temperature. The moisture content was calculated by subtracting the initial weight from the final weight after drying, expressed as a percentage of the initial weight. The water activity (aw) was measured at 25 °C with AW Sprint TH500 (Novasina, Pfaffikon, Switzerland). The color parameters of powder were determined using a colorimeter (CM-5, Konica Minolta Optics Inc., Tokyo, Japan).

### 2.3. Bulk and Tapped Density, Hausner Ratio (HR), Carr Index (CI), Wettability, and Water Dissolubility

The bulk density, tapped density, HR, and CI values were measured [51], with slight modifications. An amount of powder was gently loaded into a 50 mL tared graduated cylinder and weighed. The volume read directly from the cylinder was then used to calculate the bulk density (ρ*_bulk_*) according to mass/volume relationship. For the tapped density (ρ*_tapped_*), the cylinder was tapped by hand 200 times. The volume was then read and used for calculation. The HR and CI were calculated using the bulk and tapped densities according to the following equations:

(1) $$\mathrm{HR}\;=\;\frac{\rho_{tapped}}{\rho_{bulk}}$$

(2) $$\mathrm{CI}=\frac{{(\rho }_{tapped}-\rho_{buk})}{\rho_{tapped}}\times 100$$

For wettability analysis [52], with slight modifications, 100 mL of distilled water at room temperature was poured into a 250 mL beaker. A metal sieve was placed at the top of the beaker. Then, 0.2 g of powdered sample was placed in the metal sieve, and the powder was manually passed through the sieve using a spoon and dispersed onto the water surface while the stop watch was started at the same time. At the end, the time necessary for the powder to become completely wet (visually determined as when all the powder particles penetrated the surface of the water) was recorded.

The dissolubility of the powder (WSM, %) was measured following the method of Aliakbarian et al. [53] with minor modifications. Then, 0.2 g of powder was dispersed in 2.4 mL of distilled water in a 14 mL centrifuge tube, followed by vortexing for 5 min. Afterwards, the mixture was centrifuged for 3000 rpm for 30 min at 4 °C, and the supernatant was collected and dried at 105 °C for 6 h. WSM was determined using the following equation.

WSM = (DWS/DWM) × 100 (3)

DWS (g) is mass of the supernatant, and DWM is the mass of the powder.

### 2.4. Particle Size and Zeta Potential Analyses

The particle size distribution of the powders was measured with laser diffraction spectrometry (LDS) using a Malvern Mastersizer 2000 Hydro MU system (Malvern Instruments Ltd., Worcestershire, UK). Measurements were conducted at room temperature using propan-2-ol, which does not dissolve MD, as the dispersant medium under standard obscuration and alignment conditions. The mean diameter was expressed as the volume-weighted mean diameter D4,3. The particle size parameters (D*v*_,0.1_; D*v*_,0.5_; D*v*_,0.9_) and span values (D*v*_,0.9_ − D*_v_*_,0.1_/D_v,0.5_) were also recorded.

The particle size and zeta potential measurements of curcumin–lecithin complexes after reconstitution in distilled water were performed using a Litesizer 500 Dynamic light scattering (DLS) instrument (Anton Paar GmbH, Graz, Austria). The powders diluted 1000 times with water were vortexed for 1 min and transferred into a quartz cuvette, and the hydrodynamic diameter (z-average) was measured. For zeta potential analysis, the electrophoretic light scattering (ELS) method was used with the Smoluchowski approximation (Henry factor: 1.50) in Univette cuvettes.

### 2.5. Determination of Curcumin Content Using HPLC and LC/MS/MS

The quantification of curcumin in powders was carried out using high-performance liquid chromatography (HPLC) based on the method of Martins et al. [54] with modifications. The analysis was performed on a Thermo Vanquish HPLC-UV system, with the UV detector set at a wavelength of 425 nm. Separation was achieved using a C18 reversed-phase column (100 mm × 4.6 mm, 5 µm) maintained at 25 °C. The mobile phase consisted of 100% acetonitrile and 20 mM phosphoric acid solution, mixed in a 40:60 (*v*/*v*) ratio and delivered in isocratic mode at a flow rate of 1.0 mL/min. The injection volume was 5 µL. The working concentrations of curcumin 0.016, 0.064, 0.096, 0.128, 0.160, and 0.192 mg/mL were used to prepare the calibration curve, yielding y = 48.3132x − 0.1645 and an R^2^ value of 0.9970.

Quantitative analysis of curcumin after in vitro digestion analysis was performed using a 6475 Triple Quadrupole LC/MS/MS system (Agilent Technologies, Santa Clara, CA, USA) equipped with an electrospray ionization (ESI) source operating in positive ion mode. Chromatographic separation was achieved on a C18 reversed-phase column (100 × 2.1 mm, 1.8 µm) maintained at 40 °C. The mobile phase consisted of solvent A: 0.1% formic acid in water and solvent B: 0.1% formic acid in acetonitrile, delivered in isocratic mode at a flow rate of 0.3 mL/min, with a 40:60 (A:B) ratio. The injection volume was 5 µL. Data acquisition was carried out in Multiple Reaction Monitoring (MRM) mode. The precursor and product ion transitions for curcumin were monitored at *m*/*z* 369 → 177, with optimized fragmentor voltage and collision energy settings. The system was controlled and processed via Agilent MassHunter software version 12.

### 2.6. Entrapment Efficiency (EE%) and Loading Ratio of Curcumin

The methods of Peanparkdee and Yooying [55] and Zhang et al. [56] were utilized with minor modifications. The powders were suspended in distilled water to determine the amount of curcumin interacted with MD (m_1_). Then, 50% aqueous ethanol (the solvent of the feed solution) was used to dissolve all matrix and determine the total amount of curcumin in powder (m_2_). The supernatants were analyzed using HPLC (2.5). The entrapment efficiency and loading ratio of curcumin in complexes were calculated as follows:

(4) $$EE\%=\frac{m_{2}-m_{1}}{m_{1}}$$

(5) $$Loading\;ratio\;\%=\frac{m_{2}}{g\;powder}\times 100$$

### 2.7. Thermal Stability

The powders suspended in distilled water were incubated at 65 °C and 90 °C. Aliquots were collected at 0, 2, 4, and 6 h, and curcumin concentrations were quantified using HPLC as described in Section 2.5.

### 2.8. Stability in Simulated Gastrointestinal Fluids

Stock solutions of test compounds and positive controls (1 mM in DMSO) were prepared, with chlorambucil serving as the positive control for simulated intestinal fluid (SIF) and erythromycin for simulated gastric fluid (SGF). The SGF and SIF were prepared according to Dressman et al. [57] and Wu et al. [58] with minor modifications by dissolving 10 g pepsin in 800 mL ultrapure water, adding 16.4 mL diluted HCl (prepared by diluting 234 mL concentrated HCl to 1 L with water), and adjusting the final volume to 1 L (pH ~1.2). The SIF was prepared by dissolving 6.8 g KH_2_PO_4_ in 500 mL water, adjusting to pH 6.8 with 0.1 M NaOH, adding 10 g of trypsin, and bringing the final volume to 1 L with ultrapure water. For the stability assay, 199 μL of pre-warmed (37 °C, 15 min) SGF or SIF was aliquoted into each well of an incubation plate. Then, 1 μL of sample or control compound (1 mM) was added to achieve a final concentration of 5 μM (0.5% DMSO). The plate was incubated at 37 °C, and reactions were stopped at 0 and 120 min by adding 600 μL cold acetonitrile:water (1:1, *v*/*v*) containing internal standard (desipramine). Samples were vortexed for 10 min and centrifuged at 4000 rpm for 30 min. The supernatants were analyzed using LC/MS/MS as described in Section 2.5.

### 2.9. Scanning Electron Microscopy (SEM) and Transmission Electron Microscopy (TEM)

The morphology of the powders was assessed by a scanning electron microscope (SEM). Samples were immobilized in an appropriate support with a conductive adhesive tape, coated with palladium-gold in a sputter coater (SC7620, Quorum Technologies, East Sussex, UK), and examined with a scanning electron microscope (JSM-7001F, Jeol, Japan) at a voltage of 10 kV and a magnification ranging from 500 to 2000 times.

The microstructures of powders after reconstitution in water were investigated using a transmission electron microscope (JEM-2100, Jeol, Tokyo, Japan). The sample was suspended in distilled water and sonicated for 5 min. A few drops were dispersed on thin carbon coated-TEM micro grids and allowed to dry at room temperature before capturing the TEM images [20].

### 2.10. Differential Scanning Calorimetry (DSC), Fourier Transform Infrared Spectroscopy (FTIR), and X-Ray Diffraction (XRD) Analysis

The thermal behaviors of powders were analyzed with a DSC (DSC-60 Plus, Shimadzu, Japan). Samples of around 1.4 mg were sealed in an aluminum pan and heated from 25 to 250 °C at a speed of 10 °C/ min [38]. The control empty pan under the same heating conditions was taken as a reference.

FTIR analysis was performed to identify the chemical groups and bonding arrangement of the different components in powders. A spectrometer (Nicolet Summit FTIR Spectrometer, Thermo Fisher Scientific, Waltham, MA, USA) equipped with an Everest ATR (attenuated total reflectance) optical base and fitted with an XR Diamond Crystal Plate was used. Measurements were taken in a range of wavenumber between 4000 and 400 cm^−1^ and 32 scans per sample.

X-ray diffraction (XRD) patterns of powders were assessed according to Yang et al. [35] using high resolution diffractometer (D8 DISCOVER, Bruker, Karlsruhe, Germany) fitted with X-ray generator. Measurements were taken in the diffraction angle (2θ) range of 5–50° at a scanning speed of 5°/min. A step size of 0.03°, time per step of 60 s, voltage of 40 kV, and current of 40 mA were utilized.

### 2.11. Statistical Analysis

All analyses were performed in triplicate with triplicate samples. The statistical analysis was carried out using SPSS version 20 (IBM, Armonk, NY, USA). The data were provided as the mean ± SD. Significant differences between means were evaluated using one-way analysis of variance (ANOVA) at *p* ≤ 0.05, followed by Tukey’s post hoc test.

## 3. Results and Discussion

### 3.1. Characterization of Spray-Dried Lecithin–Curcumin Complexes

In phytosomes, the lecithin-to-polyphenol mass ratio typically ranges from 1:1 to 6:1 [44,55,56]. In this study, lecithin-to-curcumin mass ratios of 2.5:1 and 5:1 were adopted to achieve high curcumin content in the final powder while ensuring the desired solubility of lecithin, curcumin, and MD in the same aqueous ethanol solvent system. Although spray-drying was not applied in previous studies, where lecithin–polyphenol complexes were stabilized with various polymers and solidified by freeze-drying, the polymer-to-phytosome mass ratio varied as follows: 2:1 to 1:2 for PVP [38], 4:1 to 1:1 for silica [35], 9:1 to 3:1 for hydroxyapatite [44], 2.7:1 for CMC, and 4.4:1 for alginate [43]. In another study, Nguyen et al. [50] prepared lecithin–curcumin complexes (20:1, *w*/*w*) stabilized with gum Arabic using spray-drying, employing polymer to lecithin–curcumin complex mass ratios from 1:1 to 5:1. The 3:1 ratio was selected as optimal based on solubility, encapsulation efficiency, and curcumin loading. Although product yield values were not reported, no significant differences were observed among the formulations, with total solid concentrations in the spray-drying feeds ranging from 5 to 15% (*w*/*v*).

In prior work, we evaluated whey protein concentrate (WPC) and maltodextrin (MD) as carriers for olive mill wastewater–lecithin complexes and observed that WPC led to clear phase separation and reduced the yield, likely attributable to its polyelectrolytic nature [59]. Considering that lecithin has an anionic character, MD was selected as the carrier in this study because it is a hydrophilic, nonionic, and non-adsorbing polysaccharide. For the present study, a high content of lecithin–curcumin complex (phytosome) was desired in spray-dried powders; therefore, a lower amount of carrier was preferable. Preliminary experiments demonstrated that reducing the MD-to-lecithin ratio below 1:1 resulted in phase separation. Consequently, in the spray-drying experiments, the MD to lecithin was maintained above this threshold, corresponding to approximately 10–20% (*w*/*v*) total solid content in the feed solution. Additionally, MD is essential as a carrier in spray drying, and our previous experience [59] showed that higher lecithin content in the feed caused substantial stickiness issues within the drying chamber, which led to lower product yields.

The product yields of our samples ranged from 70 to 86%, comparable to the results reported by Le-Tan and Nguyen et. [60], who achieved yields approximately 75 to 88% using different ultrasonication powers for homogenizing lecithin–curcumin complexes with gum Arabic and maintaining a constant polymer-to-complex mass ratio of 3:1 during spray drying. In our study, increasing the carrier-to-lecithin–curcumin ratio (formulation F1) led to a significant improvement in spray-drying yield, reaching 86.33 ± 3.51%. The high mobility and surface activity of lecithin facilitates its rapid migration to the particle surface during drying. Additionally, relatively low melting temperature (50–65 °C) of lecithin makes it plastic and adhesive [61] under spray-drying conditions, which can increase stickiness in the drying chamber and subsequently decrease powder yield when higher concentrations are used in the feed. Moreover, increased lecithin content is expected to raise the feed viscosity, which may slow evaporation rates [62].

Moisture contents in spray-dried powders below 5% could be classified as microbiologically safe for long-term storage. Lower moisture content also limits the role of water as a plasticizer, thereby minimizing powder caking during storage [62]. Lecithin-free powders (MD-curcumin) produced under the same conditions exhibited lower water activity (0.16–0.27) and moisture content (0.47–1%), with higher carrier amounts corresponding to lower values. In contrast, lecithin–curcumin powders showed moisture contents ranging from 3.88 to 4.30%, where a higher carrier ratio tended to reduce lower moisture content (*p* > 0.05), and the water activity values were around 0.47 (Table 1). Due to its surface-active nature, layering of lecithin on the particle surface during drying likely enhanced water binding and retention [63], contributing to higher moisture content and water activity values. The moisture contents of our samples were higher than the values reported previously [60], likely due to differences in carrier polymers and spray-drying process conditions. For comparison, Gopi et al. [20] reported a moisture content of 7.9% in powders prepared from lecithin, patented curcumin, and cellulose nanofiber blended in a 1:1:1 mass ratio and processed using high-pressure homogenization and spray-drying, which can be ascribed to the elevated lecithin content.

Curcumin loading in lecithin complexes has previously been reported as 1.15%, increasing to 4 to 11% depending on the type of stabilizing polymers employed [46]. For gum Arabic stabilized spray-dried lecithin–curcumin complexes, loading ranged from 0.8 to 2.4% depending on the carrier ratio [50]. In contrast, Gopi et al. [20] reported a curcumin loading of 14.26% in lecithin-nanofiber spray-dried powders, while chitosan microspheres loaded with curcumin phytosome showed a loading rate of 2.67% [56]. Resveratrol–lecithin complexes stabilized by fumed silica via freeze-drying achieved 11.86% loading of active compound, and berberine–lecithin complexes carried by SiO_2_ and TPGS (D-α-tocopheryl polyethylene glycol 1000 succinate) using vacuum-drying showed loadings between 6.1 and 10.72% depending on the carrier ratio [45]. In the present study, the theoretical curcumin loading in the spray-dried curcumin–lecithin complexes was around 9% for formulations F1 and F3 and 16% for F2. Upon the solubilization of powders and subsequent curcumin quantification, the loading rate ranged from ranged from 8.21 to 14.80% (Table 1), with the differences likely arising from stresses that occurred during spray-drying.

Entrapment efficiencies of curcumin in the lecithin complexes were 93.97, 93.05, and 86.50% for F1, F2, and F3, respectively. These values were higher than the 30–70% range reported by Nguyen et al. [50] for spray-dried lecithin–curcumin particles. Zhang et al. [56] reported that 90.81% of curcumin could be complexed with phospholipids, while Peanparkdee et al. [55] found greater than 98% entrapment of vitexin in soybean and egg yolk phosphatidylcholine. In our samples, the surface curcumin content was highest in F3, which had the lowest curcumin-to-lecithin ratio, likely due to reduced interaction and complex formation. The other two formulations with constant lecithin-to-curcumin ratios showed no significant change depending on the carrier ratio.

The spray-dried powders were analyzed using laser light diffraction to determine the particle size distribution. The volumetric mean particle size (D_4,3_) of the curcumin phytosome powders ranged from 12.92 to 69.45 µm, which were comparable to the particle size range (29 to 42 µm) of spray-dried curcumin–lecithin particles reported by Nguyen et al. [50], in which ultrasonic homogenization was performed prior to spray-drying. Notably, none of our samples exhibited a unimodal particle size distribution, as all demonstrated span values exceeded 2, indicating a broad size distribution. Increasing the carrier-to-lecithin ratio from 1 to 3 (comparing F3 to F1) resulted in a significant reduction in particle size with volumetric mean values decreasing from 36.81 to 12.92 µm. Conversely, at the constant carrier-to-lecithin ratio (1:1), increasing the curcumin content led to larger particle size, reaching up to 69.45 µm, and was associated with the presence of larger aggregates (Table 1).

**Figure 1:**
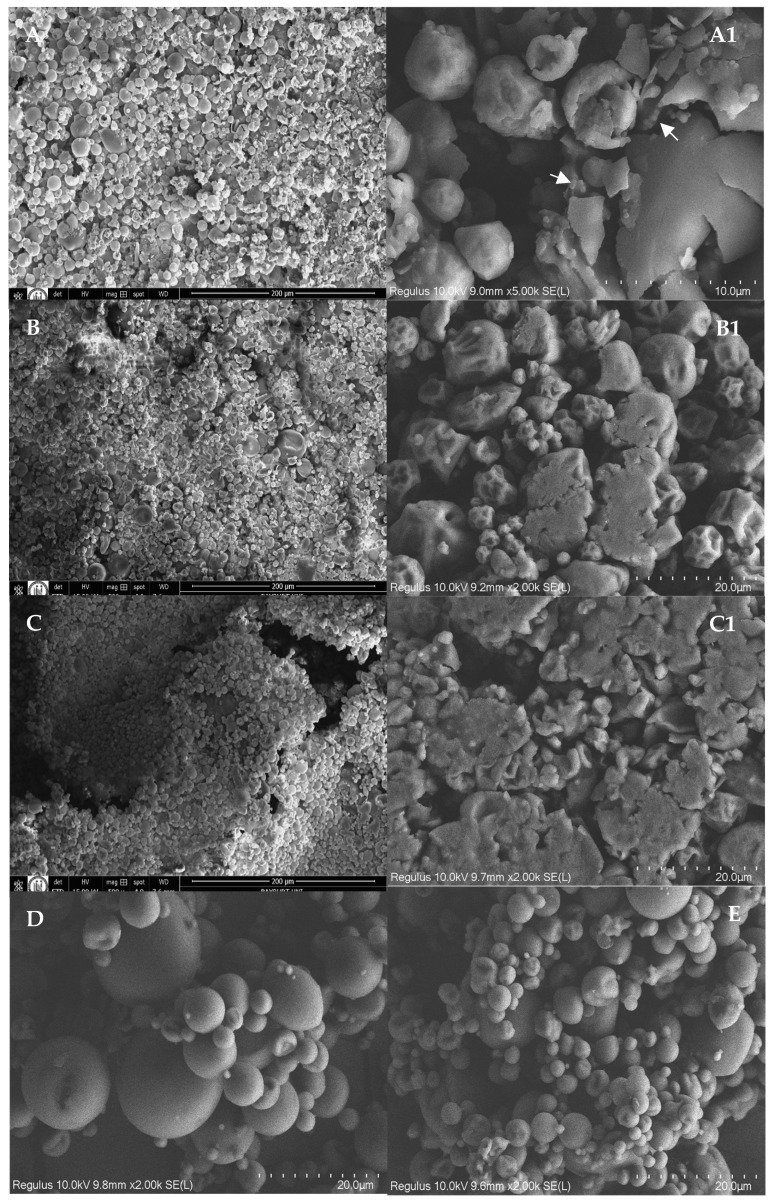
SEM images of F1 (**A**,**A1**), F2 (**B**,**B1**), F3 (**C**,**C1**), MD15 (**D**), and MD5 (**E**). The mass ratios of lecithin:curcumin:maltodextrin were 5:2:15; 5:2:5, 5:1:5, 0:1:5, and 0:1:15 for F1, F2, F3, MD5, and MD15, respectively.

Wettability is defined as the time (in seconds) required for a specific quantity of powder to penetrate the still surface of water. It reflects the ability of a powder to absorb water on its surface and become wet. The wettability of powder particles is influenced by factors such as the surface activity of the particles, surface area, surface charge, particle size, density, porosity, and presence of moisture-absorbing substances [64]. When the surface becomes more hydrophilic, water interacts predominantly with hydrophilic regions, which accelerates water spreading and reduces the wetting time [65]. Among all powder formulations (Table 2), those without lecithin showed elevated wettability, indicating that the lecithin layer on the particle surface increased the time required for wetting. Furthermore, between F2 and F3, which differ only in the curcumin content, wettability was further decreased with higher amounts of hydrophobic curcumin. However, among lecithin-free powders, MD5 exhibited better wettability despite having more curcumin, likely due to its more agglomerated structures as seen in SEM images (Figure 1). For example, in spray-dried milk powders, larger particles and aggregates tend to wet more easily than smaller particles because they create larger spaces between individual particles, facilitating water penetration [64].

Dissolubility is defined as the ability of powders to form solution or suspension in water, with higher dissolubility being desirable, especially when powders are used as an additive in food products. In this study, powder dissolubility ranged from 72 to 85%, with higher curcumin loading corresponded to lower dissolution (Table 2). Nguyen et al. [50] reported that varying the lecithin-to-gum Arabic mass ratio from 1:1 to 5:1 changed the dissolubility of spray-dried powder from 56 to 84%. The reduced molecular crystallinity of curcumin and the amphiphilic nature of produced phytosomes can be cited as the main reasons for the increase in dissolubility in water. The interaction with lecithin may reduce the lattice energy of the crystalline curcumin, allowing solvent molecules to penetrate and dissolve the compound more effectively [55].

The bulk and tapped densities, together with the Carr compressibility index (CI) and Hausner ratio (HR), are parameters used to predict the interparticle interactions and flow properties of powders. The bulk density is an important characteristic of powder/granule foodstuffs in terms of their storage and ease of transportation. Lecithin–curcumin complex powders showed significantly lower bulk and tap densities compared to lecithin free-curcumin powders (Table 1). However, this difference was reflected in CI and HR values only in formulation F1, which had higher the carrier-to-lecithin ratio. In powders with good flowability, the bulk and tapped densities are close in value, whereas a larger difference between these densities indicates poorer flow due to stronger interparticle interactions, such as particle bridging. Formulation F1 demonstrated fair flowability, as pharmaceutical powders with CI values between 16 and 20 and HR values between 1.12 and 1.18 are classified as having fair flow, while powders with CI values between 26 and 31 and HR values between 1.35 and 1.4 are considered to have poor flowability [66]. The color values represented by lightness (L), redness (a), and yellowness (b) values were indicative of the MD carrier ratio and curcumin loading. The lecithin-free sample of MD5, in which the MD-to-curcumin ratio was the lowest, yielded the highest redness and lowest lightness values. Among lecithin–curcumin complex powders, similarly the sample with higher MD had higher L and lower a values (Table 2).

### 3.2. Morphology of Spray-Dried Lecithin–Curcumin Complexes

SEM images of samples (Figure 1) revealed distinct morphology differences among the samples. Lecithin-free powders (MD15 and MD5) exhibited smooth surfaces, without apparent dents or wrinkles (Figure 1D,E). However, MD5 displayed larger differences in particle sizes and higher particle cohesion, evidenced by grape bunch-type agglomerates. In contrast, lecithin–curcumin complexes showed variations in surface morphology primarily related to the degree of particle agglomeration and surface topography. Compared to formulations F2 and F3, which had a lower carrier ratio, the F1 formulation (Figure 1A) consisted of more spherical and discrete particles, with smoother surfaces, although some larger particles were fractured. The interiors of those fractured particles revealed subglobose structures (white arrows), likely aggregated lecithin–curcumin under the shell provided by MD. Formulations F2 and F3 with only curcumin content were different. These formulations could also be characterized by clusters of adhered particles, with more pronounced surface deflation and crushed morphologies.

**Figure 2:**
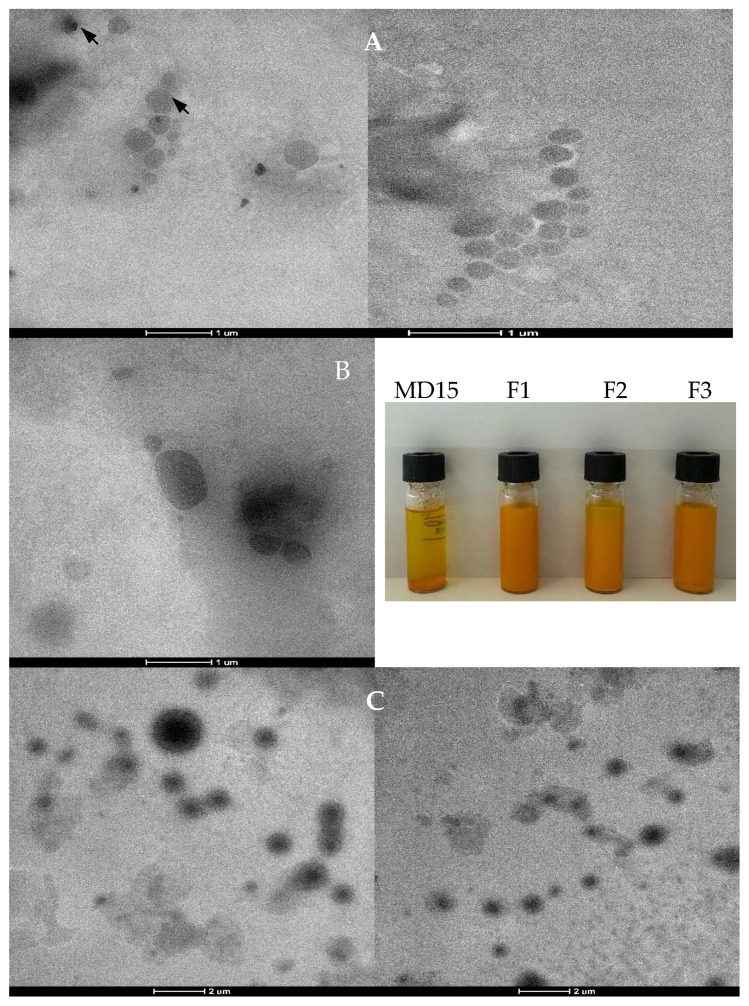
TEM images F1 (**A**), F2 (**B**), and F3 (**C**) powders after reconstitution in water. Inset shows pictures of the reconstituted powders after 30 days storage under room conditions. The mass ratios of lecithin:curcumin:maltodextrin were 5:2:15; 5:2:5, and 5:1:5 for F1, F2, and F3, respectively.

TEM images of reconstituted lecithin–curcumin complexes in water (Figure 2) revealed spherical or ellipsoidal particles with a core–shell structure. The formation of curcumin–phospholipids complexes did not alter the amphiphilic and water dispersibility properties of phospholipids. In TEM analysis, denser regions hinder electron transmission, appearing in dark areas that likely correspond to the higher occurrence of curcumin. In the F1 powder, which had a higher carrier content, these darks areas could have been shielded, presumably by the MD shell surrounding the core. The average sizes of reconstituted powders were 1.32, 1.83, and 1.25 µm for F1, F2, and F3, respectively (Table 3). The F2 formulation exhibited the largest particle size, which could be attributed to its higher curcumin loading. It has been reported that binding of curcumin perturbs the packing characteristics of the phospholipid bilayer, resulting in a looser structure [37].

### 3.3. DSC, FTIR, and XRD Analysis

**Figure 3:**
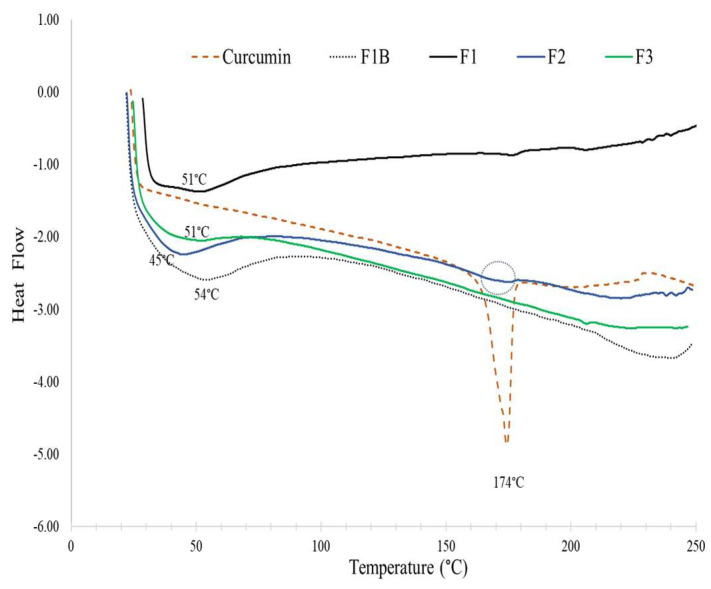
DSC thermograms of curcumin, F1, F2, and F3. The mass ratios of lecithin:curcumin:maltodextrin were 5:2:15; 5:2:5, and 5:1:5 for F1, F2, and F3, respectively. F1B is the curcumin-free formulation of F1.

Differential scanning calorimetry (DSC) thermograms of samples given in Figure 3 may provide insights into the melting behavior of the curcumin and its encapsulation within the formulations. The first endothermic peak observed in lecithin–curcumin complexes corresponded to the melting of acyl chains in the phospholipid bilayer from the rigid gel phase to the fluid liquid crystalline phase [37]. The lecithin–MD blank powder (F1B) yielded a melting peak at 54 °C, which decreased to 51 °C in formulation F1 upon complexation with curcumin. In F2 formulation, where the curcumin concentration was twice that of F3, this peak further decreased to 45 °C. The interaction of curcumin with the polar part of phospholipid molecules makes the long hydrocarbon tail of phospholipids turn freely and ‘envelop’ the polar head of phospholipids containing the curcumin molecule, yielding a decrease in the sequence of the phospholipid hydrocarbon chains and reducing the phase transition temperature [42]. This trend was also consistent with previous findings where the increasing curcumin concentration in liposomes resulted in a gradual reduction of the phase transition temperature from 51.2 to 47.7 °C, reflecting the disruptive effect of curcumin binding on phospholipid packing, thus yielding a looser and more flexible bilayer structure [37].

The DSC thermogram of pure displayed a sharp melting at 174 °C, and only F2 powder showed a small broad melting endotherm in this region, attributable to its higher curcumin loading. The broadening and disappearance of the curcumin melting peak in the lecithin complexes indicate the formation of amorphous structure and molecular interaction of curcumin with lecithin and MD. Similar reductions and alterations in the curcumin melting peak have been reported previously [42,56].

**Figure 4:**
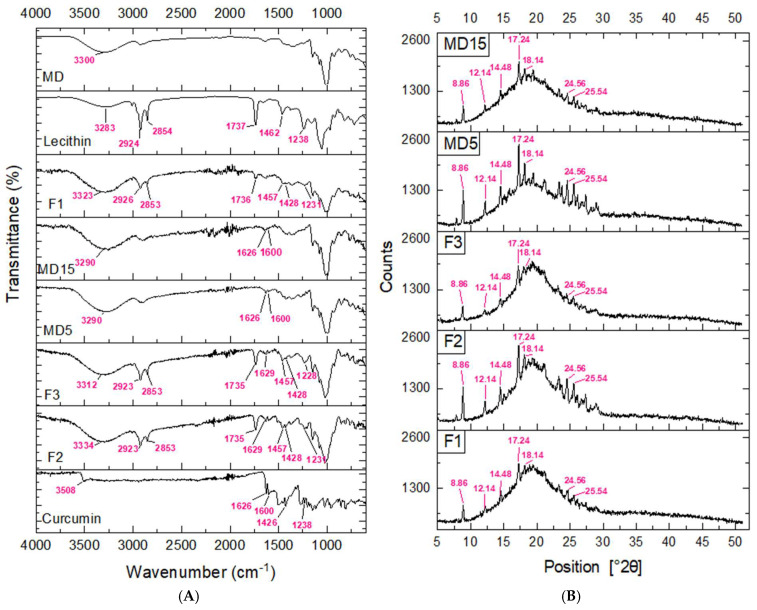
FTIR spectra (**A**) and XRD patterns (**B**) of curcumin, F1, F2, and F3. The mass ratios of lecithin:curcumin:maltodextrin were 5:2:15; 5:2:5, 5:1:5, 0:1:5, and 0:1:15 for F1, F2, F3, MD5, and MD15, respectively. MD: Maltodextrin.

The ATR-FTIR spectra of curcumin, lecithin, MD, and powder samples are given in Figure 4A. Characteristic O–H stretching vibrations were identified for curcumin, lecithin, and maltodextrin at 3508, 3283, and 3300 cm^−1^, respectively. In curcumin, this band reflects the phenolic O–H group. When curcumin was incorporated into maltodextrin without the use of lecithin, the O–H band appeared at a lower absorbance (3290 cm^−1^), while its presence led to shifts to higher wavenumbers—3323, 3334, and 3312 cm^−1^ air spray-dried formulations of F1, F2, and F3, respectively. These spectral changes indicate the establishment of hydrogen bonds between hydroxyl groups of curcumin, maltodextrin, and the phosphate (P=O) groups of lecithin. Such interactions stabilize curcumin by facilitating intermolecular hydrogen bonding [22,55]. Similar alterations in spectra phyotosomes loaded with resveratrol were reported previously [67].

Lecithin exhibited bands at 2924 and 2854 cm^−1^ corresponding to asymmetric and symmetric C–H stretching vibrations, characteristic of its long fatty acid chains. This region is sensitive to conformational changes in lipid acyl chains and is used to monitor information associated with the overall degree of ordering and packing of the lipid bilayer during interactions with membrane-active molecules [68]. This symmetric CH_2_ stretching band was slightly decreased to 2853 cm^−1^ in all phytosome-loaded powders. However, the asymmetric stretching band (2924 cm^−1^) decreased to 2923 cm^−1^ in F2 and F3, but increased to 2926 cm^−1^ in F1 sample in which the carrier to phytosome ratio was higher. Such low-frequency shifts in these hydrophobic regions suggest hydrophobic interactions between curcumin and lecithin lipid tails. Specifically, a shift to lower wavenumbers reflects a more disordered lipid acyl chain arrangement, indicating increased bilayer fluidity; conversely, shifts to higher wavenumbers suggest increased membrane order [68,69].

A strong peak at 1737 cm^−1^ lecithin was attributed to symmetric C=O ester vibrations of the polar head of the phospholipid and provides insights about hydrogen bonding to the ester carbonyl groups. Variations in this peak within lecithin–curcumin complexes—such as a shift to 1735 cm^−1^, peak broadening, and reduced intensity—suggest changes in hydrophobic packing between curcumin and phospholipid molecules and hydrogen bonding to the ester carbonyl group, further reflecting the integration of curcumin within the lipid phase. The absence of this peak in MD–curcumin powders confirms its specificity to phospholipid components. Curcumin alone displays prominent peaks at 1626 cm^−1^ and 1600 cm^−1^, attributed to C=C and C=O stretching in the conjugated interring chain and aromatic rings. This band at 1626 cm^−1^ was maintained in lecithin-free curcumin powders, particularly MD5 in which the carrier ratio was low. However, in curcumin phytosome-loaded powders (F2 and F3, in which MD carrier ratio was low), it was displaced to 1629 cm^−1^ at a lower intensity. This could result from the hydrophobic and altered molecular environment resulting from the association of curcumin’s conjugated system with the nonpolar regions of lecithin. The band seen in lecithin at 1462 cm^−1^ was related to bending vibrations of CH_2_ manifesting at 1457 cm^−1^ in F2 and F3 and at a lower intensity in F1. This could be related to carrier and lecithin interactions. The characteristic peak at 1426 cm^−1^ of curcumin related to the C=O stretching was detected at 1428 cm^−1^ in lecithin–curcumin powders. In lecithin, the band at 1238 cm^−1^ was due to the PO_2_ asymmetric vibration, and this peak was displaced to 1231 cm^−1^ in F1 and F2 and 1228 cm^−1^ in F3 formulations. A decrease in the wavenumber of PO_2_ may indicate that curcumin promotes the formation of hydrogen bonds with the phosphate groups [68].

The curcumin molecule was demonstrated to be anchored inside the phospholipid bilayer through the hydrogen bonding of −OH groups of the phenolic rings of curcumin with the head groups of phospholipids and the hydrophobic interactions of the aromatic rings of curcumin with phospholipid acyl chains [37]. The band displacement to a higher or lower wavelength indicates the existence of an interaction between the functional groups that existed in the ingredients. The observed ATR-FTIR spectral changes demonstrate both hydrogen bonding and hydrophobic interactions that could make the formulations more robust and offer improved physicochemical properties for curcumin delivery.

Free curcumin was reported to display its characteristic XRD peaks in the range of 5° to 30°, including prominent peaks at 8.86, 12.14, 14.48, 17.24, 18.14, 24.56, and 25.54 [22,70]. When comparing the diffraction peaks of lecithin-free powders and curcumin–lecithin complex powders, the sharp peaks associated with curcumin crystallinity, which was particularly evident in MD5, were significantly reduced in the F1 and F3 samples (Figure 4B). The low diffraction intensity of curcumin samples suggests that curcumin was either molecularly dispersed in a phospholipid matrix or in a predominantly amorphous form. Similar results were supported by the previous studies performed with the phospholipid complexes [71].

### 3.4. Thermal Stability

**Figure 5:**
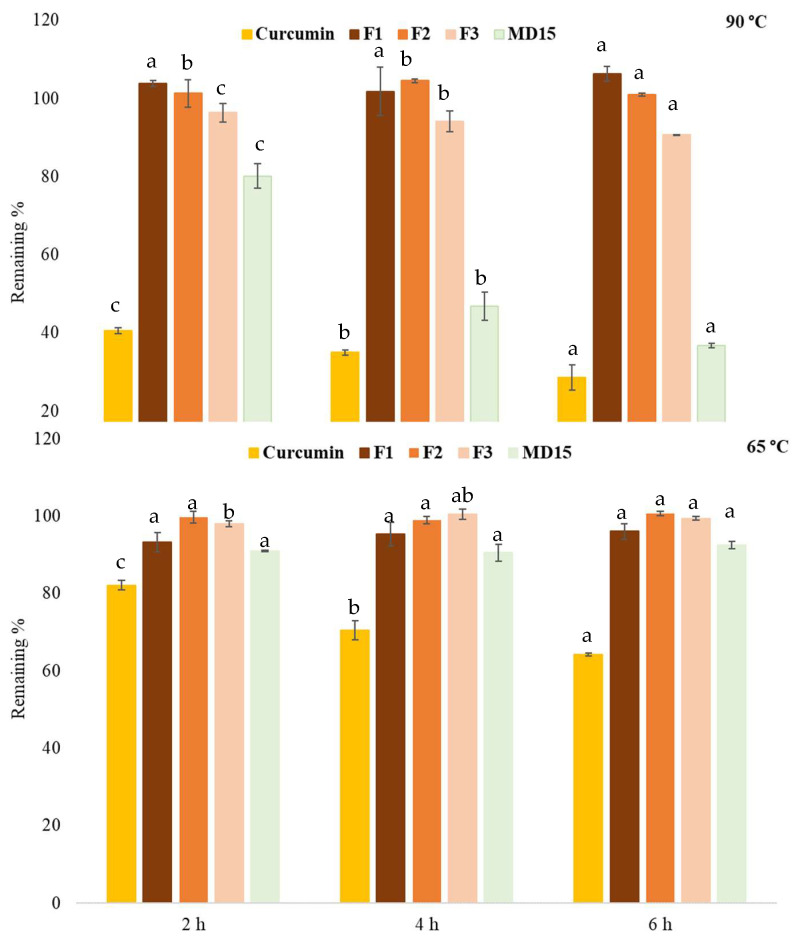
Thermal stability of curcumin and F1, F2, and F3. Values for same samples among incubation hours with different superscript letters are significantly different (*p* < 0.05). The mass ratios of lecithin:curcumin:maltodextrin are 5:2:15; 5:2:5, 5:1:5, and 0:1:15 for F1, F2, F3, and MD15, respectively.

The aqueous dispersions of powders were incubated at 90 and 65 °C up to 6 h, and curcumin content was compared to the initial amount (Figure 5). At the end of incubation period at 65 °C, free curcumin showed around 35% of degradation, while all powder formulations, including lecithin-free M15, provided greater than 90% protection. Free curcumin showed around 70% of degradation at 90 °C after 6 h. In contrast, formulations F1 and F2 were both retained the initial loaded amount, and F3 gradually showed around 10% of reduction. MD15 showed a gradual reduction with incubation time and reached an approximately 40% reduction in its curcumin content. Elevated temperatures can induce the transition of the phospholipid bilayer from the highly ordered gel phase to the fluid liquid crystalline phase. Additionally, the partial decomposition of hydrogen bonds at higher temperatures may compromise the structural integrity of phytosome, leading to curcumin leakage and subsequent degradation reactions [37].

### 3.5. Stability Under Simulated Digestion Conditions

**Figure 6:**
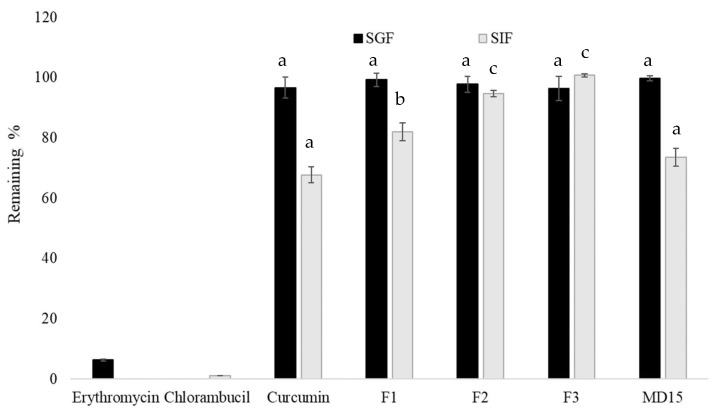
Stability of curcumin, F1, F2, and F3 under simulated digestion conditions. Values for the same SIF conditions that do not share a superscript letter are significantly different (*p* < 0.05). The mass ratios of lecithin:curcumin:maltodextrin were 5:2:15; 5:2:5, 5:1:5, and 0:1:15 for F1, F2, F3, and MD15, respectively.

Digestive stability is an important index to evaluate the bioavailability of bioactive compounds. The samples, including free curcumin, were incubated at acidic pH 1.3 for 2 h, representing the gastric pH, and incubated at pH 6.9 for 2 h, representing the intestinal pH conditions. Erythromycin and chlorambucil, positive controls for gastric and intestinal buffer, were also subjected to the incubation under the same conditions. As shown in Figure 6, both positive controls almost completely degraded in the related medium. None of the samples, including free curcumin, did not show any degradation after 2 h of incubation at gastric buffer. On the other hand, around 32% of free curcumin was degraded after 2 h of incubation at intestinal buffer medium (pH 6.9). It was previously reported that the decomposition was pH-dependent and occurred faster under neutral and basic conditions. The increased stability of curcumin in acidic pH condition may be facilitated by the conjugated diene structure. However, when the pH is adjusted to neutral and basic conditions, protons are removed from the phenolic group, leading to the destruction of this structure, and this process is accelerated in the presence of the light [72]. Among the powders, MD15 exhibited similar behavior to free curcumin, with around 26% degradation. The interaction with lecithin protected the hydrolytic degradation of curcumin under alkaline media because our lecithin–curcumin complex powder formulations yielded higher stability. Here, 82 to 100% of curcumin remained after 2 h of incubation at pH 6.9, depending on the carrier ratio (MD). It has been shown that in the curcumin-loaded lecithin complexes, the curcumin molecule is not completely anchored to the inside of the phospholipid cluster, and the polar part of curcumin is located at the interface of phospholipid layer [38,46]. Therefore, when MD was adsorbed on the outer layer of phospholipid molecule, it could have also individually interacted with curcumin and lecithin, thus reducing their complexation.

Exposure of free curcumin to alkaline media increases its solubility by deprotonation of phenolic and enolic –OH groups, but simultaneous rapid hydrolytic degradation also occurs. In contrast, within phytosomes, curcumin is molecularly complexed with lecithin, forming a lipid bilayer or vesicular structure that physically surrounds and isolates curcumin from the external alkaline environment. Curcumin also establishes both hydrophobic and hydrogen bond interactions with phospholipid headgroups and tails, stabilizing the more resistant keto-enol form, and restricting molecular transformations that precede chemical degradations [73]. Under intestinal digestion conditions, bile salts can penetrate phospholipid bilayers, increasing membrane fluidity. This enhanced fluidity in turn facilitates lipase adsorption, eventually disrupting the structural integrity of the phytosome and causing the rapid release of encapsulated curcumin.

While the phytosome structure is effective in improving curcumin’s solubility and stability during intestinal digestion, recent findings underline the critical importance of the micellization efficiency of curcumin delivery systems for oral bioavailability. Flory et al. [74] compared seven curcumin delivery systems, including phytosomes, and reported a 7.5-fold increase in plasma curcumin concentrations following phytosome administration, relative to native curcumin. However, this increase was considerably less than that achieved with micellar curcumin (57-fold) and γ-cyclodextrin complexes (30-fold), differences that correlated with the micellization efficiency of the delivery systems. Specifically, phytosome formulations retained 51% curcumin stability after in vitro digestion but exhibited only 3.8% micellization efficiency. In contrast, polysorbate 80 micelles yielded 100% stability and 55% micellization efficiency, and γ-cyclodextrin complexes achieved 70% stability and 22% micellization. These results highlight the importance of not only protecting curcumin throughout digestion but also facilitating its effective incorporation into mixed micelles to maximize absorption.

## 4. Conclusions

This study clearly demonstrated that spray drying can be utilized to produce free-flowing curcumin phytosome powders using maltodextrin as a carrier with desirable technological and functional properties. The inclusion of maltodextrin significantly enhanced powder yield, improved flowability, and increased the aqueous solubility of the final product, facilitating the formation of powders suitable for industrial applications. The molecular interactions between lecithin and curcumin were reflected in the high entrapment efficiencies achieved and the predominantly amorphous structure observed, as confirmed by DSC and XRD analysis. These structural characteristics contributed directly to the improved thermal and digestive stability of the complexes as compared to free curcumin, allowing for effective protection of curcumin from degradation under both simulated intestinal pH and elevated temperature conditions. This enhanced stability is attributable to complexation within the phospholipid bilayer, which shields the curcumin molecule from external chemical and environmental stresses. FTIR spectroscopy further substantiated the successful complexation of curcumin, lecithin, and maltodextrin, while morphological analysis using electron microscopy revealed well-defined, spherical core–shell particles.

Collectively, these results position spray-dried lecithin–curcumin complexes as highly promising candidates for the formulation of functional food ingredients and dietary supplements. They offer practical solutions to the long-standing challenges of curcumin’s poor solubility, limited thermal and digestive stability, and handling difficulties linked to phytosome stickiness. Future studies should prioritize cell-based and in vivo bioavailability studies to confirm the biological efficacy of these formulations.

## Figures and Tables

**Table 1:** The yield, moisture content, entrapment efficiency (EE%), curcumin loading (%), and particle size of the samples.

Sample	Lec:Cur:MD	Yield (%)	a_w_	Moisture (%)	EE%	Loading Rate (%)	D_4,3_ (µm)	D_v,0.1_ (µm)	D_v,0.5_ (µm)	D_v,0.9_ (µm)	Span
F1	5:2:15	86.33 ± 3.51 ^c^	0.49 ± 0.09 ^b^	3.88 ± 0.28 ^a^	93.97 ± 0.27 ^b^	8.20 ± 0.12 ^a^	12.92 ± 1.05 ^a^	2.81 ± 0.19 ^a^	10.04 ± 1.94 ^a^	26.42 ± 4.06 ^a^	2.35 ± 0.13 ^b^
F2	5:2:5	76.00 ± 3.00 ^b^	0.47 ± 0.00 ^a^	4.04 ± 0.86 ^a^	93.05 ± 1.52 ^b^	14.06 ± 0.43 ^c^	69.45 ± 2.32 ^c^	5.91 ± 0.74 ^b^	63.39 ± 3.51 ^c^	148.24 ± 17.1 ^c^	2.25 ± 0.14 ^ab^
F3	5:1:5	70.01 ± 2.85 ^a^	0.48 ± 0.00 ^a^	4.40 ± 0.25 ^a^	86.50 ± 0.25 ^a^	8.99 ± 0.16 ^b^	36.81 ± 3.01 ^b^	5.24 ± 0.51 ^b^	34.06 ± 2.09 ^b^	72.31 ± 6.30 ^b^	1.97 ± 0.12 ^a^
MD5	0:1:5	50.37 ± 2.06	0.27 ± 0.01	1.01 ± 0.15	-		-	-	-	-	-
MD15	0:1:15	74.70 ± 1.37	0.16 ± 0.00	0.47 ± 0.06	-		-	-	-	-	-

Data are given as mean ± standard deviation. Values in the same column that do not share a superscript letter are significantly different (*p* < 0.05). a_w_, water activity; EE%, entrapment efficiency of curcumin; Lec:Cur:MD, mass ratio of lecithin:curcumin:maltodextrin; D_4,3_, volume mean diameter; D*v*_,0.1_, D*v*_,0.5_, and D*_v_*_,0.9_ represent particle sizes in the 10%, 50% and 90% quantiles of the distribution.

**Table 2:** The color, bulk, tapped densities, wettability, and solubility of the samples.

Sample	Bulk Density (g/mL)	Tapped Density (g/mL)	CI	HR	Wettability (s)	Dissolubility (%)	L	a	b
F1	0.17 ± 0.01 ^a^	0.20 ± 0.01 ^a^	15.73 ± 0.70 ^a^	1.19 ± 0.01 ^a^	69.00 ± 4.85 ^b^	84.69 ± 0.89 ^d^	81.33 ± 0.10 ^e^	10.18 ± 0.12 ^a^	42.04 ± 0.03 ^d^
F2	0.25 ± 0.00 ^b^	0.37 ± 0.00 ^b^	30.34 ± 0.60 ^b^	1.44 ± 0.01 ^b^	106.80 ± 13.22 ^d^	80.63 ± 0.53 ^b^	76.89 ± 0.08 ^c^	19.22 ± 0.11 ^c^	40.33 ± 0.07 ^c^
F3	0.28 ± 0.01 ^c^	0.40 ± 0.02 ^c^	31.35 ± 1.74 ^b^	1.46 ± 0.04 ^b^	88.00 ± 12.14 ^c^	85.07 ± 0.50 ^d^	79.87 ± 0.39 ^d^	13.08 ± 0.58 ^b^	42.18 ± 0.23 ^d^
MD5	0.42 ± 0.01 ^e^	0.60 ± 0.01 ^d^	29.63 ± 2.04 ^b^	1.42 ± 0.04 ^b^	22.00 ± 1.58 ^a^	71.79 ± 0.80 ^a^	68.36 ± 0.04 ^a^	27.49 ± 0.05 ^e^	33.90 ± 0.02 ^a^
MD15	0.36 ± 0.01 ^d^	0.63 ± 0.03 ^e^	42.57 ± 2.58 ^c^	1.74 ± 0.08 ^c^	30.80 ± 1.92 ^a^	82.06 ± 0.25 ^c^	73.66 ± 0.01 ^b^	22.41 ± 0.01 ^d^	37.63 ± 0.01 ^b^

Data are given as mean ± standard deviation. Values in the same column that do not share a superscript letter are significantly different (*p* < 0.05). HR, Hausner ratio; CI, Carr index. The mass ratios of lecithin:curcumin:maltodextrin were 5:2:15; 5:2:5, 5:1:5, 0:1:5, and 0:1:15 for F1, F2, F3, MD5, and MD15, respectively.

**Table 3:** The average sizes of reconstituted curcumin–lecithin complex powders.

	F1	F2	F3
Hydrodynamic diameter, µm	1.32 ± 0.16	1.83 ± 0.13	1.25 ± 0.13
D10, µm	0.70 ± 0.09	0.90 ± 0.08	0.54 ± 0.05
D50, µm	0.95 ± 0.19	1.15 ± 0.13	0.94 ± 0.11
D90, µm	1.38 ± 0.33	1.49 ± 0.24	1.47 ± 0.28
Span	0.63 ± 0.17	0.50 ± 0.10	0.99 ± 0.28
Zeta potential (mV)	−54.1 ± 1.04	−53.8 ± 1.05	−54.0 ± 1.01

Data are given as mean ± standard deviation. The mass ratios of lecithin:curcumin:maltodextrin were 5:2:15; 5:2:5, and 5:1:5 for F1, F2, and F3, respectively.

## Data Availability

The original contributions presented in this study are included in the article. Further inquiries can be directed to the corresponding author.
